# Functions of Sphingolipids in Pathogenesis During Host–Pathogen Interactions

**DOI:** 10.3389/fmicb.2021.701041

**Published:** 2021-08-02

**Authors:** Jian Wang, Yi-Li Chen, Yong-Kang Li, Ding-Kang Chen, Jia-Fan He, Nan Yao

**Affiliations:** ^1^State Key Laboratory of Biocontrol, Guangdong Provincial Key Laboratory of Plant Resource, School of Agriculture, Sun Yat-sen University, Guangzhou, China; ^2^State Key Laboratory of Biocontrol, Guangdong Provincial Key Laboratory of Plant Resource, School of Life Sciences, Sun Yat-sen University, Guangzhou, China

**Keywords:** infection, pathogenesis, sphingolipids, bacteria, fungi, virus

## Abstract

Sphingolipids are a class of membrane lipids that serve as vital structural and signaling bioactive molecules in organisms ranging from yeast to animals. Recent studies have emphasized the importance of sphingolipids as signaling molecules in the development and pathogenicity of microbial pathogens including bacteria, fungi, and viruses. In particular, sphingolipids play key roles in regulating the delicate balance between microbes and hosts during microbial pathogenesis. Some pathogens, such as bacteria and viruses, harness host sphingolipids to promote development and infection, whereas sphingolipids from both the host and pathogen are involved in fungus–host interactions. Moreover, a regulatory role for sphingolipids has been described, but their effects on host physiology and metabolism remain to be elucidated. Here, we summarize the current state of knowledge about the roles of sphingolipids in pathogenesis and interactions with host factors, including how sphingolipids modify pathogen and host metabolism with a focus on pathogenesis regulators and relevant metabolic enzymes. In addition, we discuss emerging perspectives on targeting sphingolipids that function in host–microbe interactions as new therapeutic strategies for infectious diseases.

## Introduction

Sphingolipids are a class of lipids that are characterized by a long-chain amino alcohol (sphingoid) backbone with an amide-bound fatty acyl chain (Heaver et al., 2018). Their structural diversity generates 1,000 of unique sphingolipids *via* the combination of various lipid headgroup and fatty acyl chains (Sud et al., 2007). Several enzymes that function in sphingolipid metabolism also act as signaling modules, and their products serve as bioeffectors. In mammalian cells, most sphingolipids studied to date regulate cellular processes such as stress responses, cell proliferation, apoptosis, cell differentiation, insulin resistance, aging, and cancer. Of the three types of pathogens (bacteria, fungi, and viruses), fungi produce their own sphingolipids for pathogenesis; by contrast, most bacteria and viruses do not synthesize sphingolipids but use host sphingolipids to promote their own virulence (Manes et al., 2003). Pathogens have evolved various strategies to use sphingolipids, thus ensuring their own survival by evading the host immune system. Therefore, understanding the means by which pathogens exploit sphingolipids could lead to the development of new therapeutic strategies to fight infectious diseases. The goal of this review is to discuss the role of microbial sphingolipids and their related metabolic enzymes in pathogenesis during their infection cycles in order to better understand host–microbe interactions mediated by sphingolipids and to explore the potential for using these sphingolipids as therapeutic targets to treat infectious diseases.

## Structure and Metabolism of Sphingolipids

**Figure 1:**
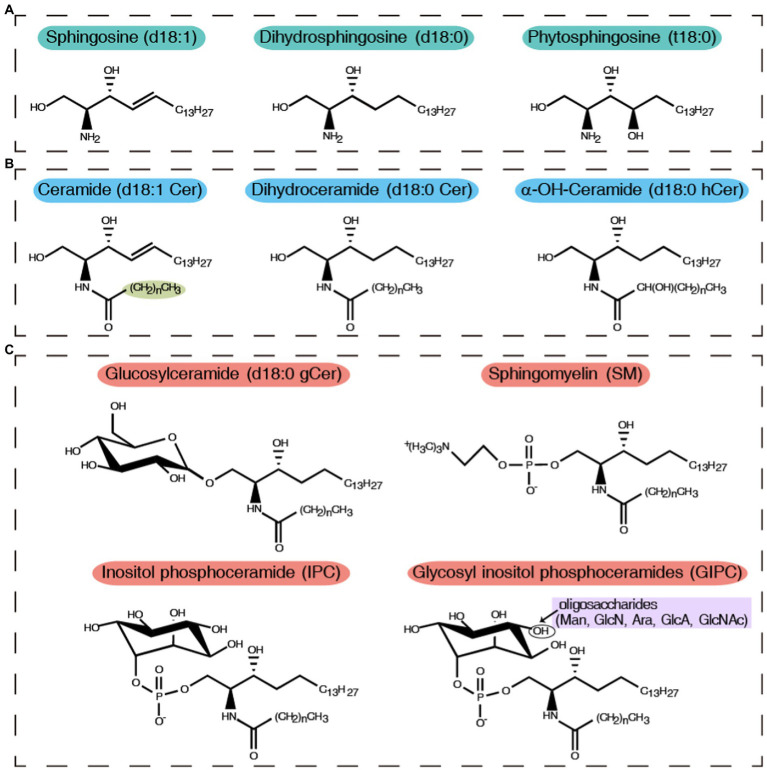
General structures, nomenclature, and abbreviated names of sphingolipids. **(A)** Sphingosine and its hydroxylation and saturation derivatives; **(B)** Ceramide and its derivatives; **(C)** Complex sphingolipids, including glucosyl sphingolipids, sphingomyelin, inositol phospho ceramide, and glycosyl inositol phospho ceramides. (CH_2_)_n_, chain length of the N-acyl sphingolipid moiety. Oligosaccharides: mannose (Man), glucosamine (GlcN), arabinose (Ara), glucuronic acid (GlcA), and *N*-acetylglucosamine (GlcNAc).

Sphingolipids are composed of a long-chain sphingoid base (LCB) backbone linked to a fatty acid (FA) and a polar head group (Figure 1). LCBs commonly contain hydroxyl groups at their C-1 and C-3 positions and an amino group at their C-2 position. LCB chain lengths in budding yeast (*Saccharomyces cerevisiae*) are C16, C18, or C20 (Ferguson-Yankey et al., 2002), and FA chain lengths range from C14 to C26. Ceramide is a long-chain fatty acid amide derivative of LCB. In yeast ceramide, the FA chain is predominantly C26 and is saturated, but it can present three distinct types of hydroxyl states (nonhydroxyl, monohydroxyl, and dihydroxyl; Dickson, 2008).

**Figure 2:**
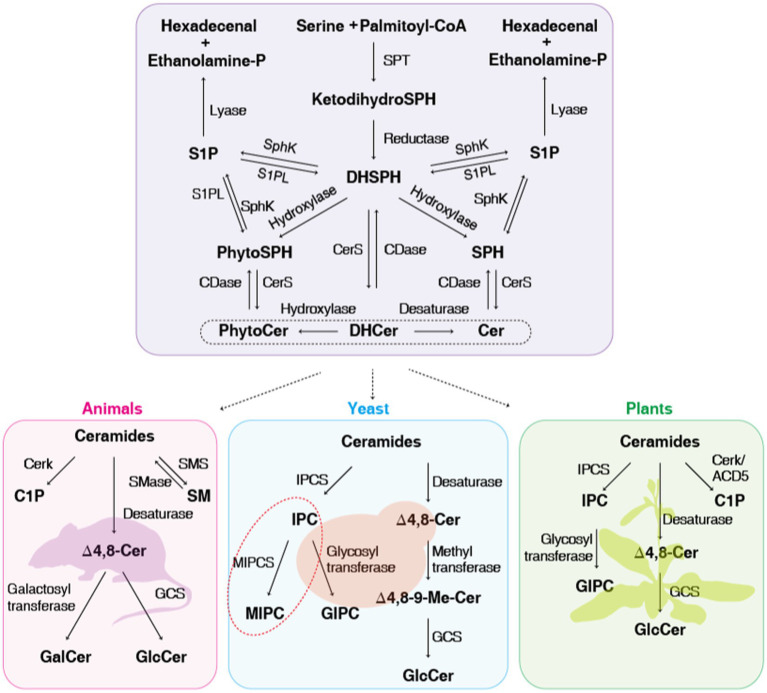
Sphingolipid metabolic pathways. A schematic representation of sphingolipids and their metabolic enzymes in fungal, plant, and mammalian systems. Metabolic intermediates and sphingolipids are shown in bold, with enzyme names next to their respective reactions. *Saccharomyces cerevisiae* only forms MIPC (red-dashed box), while other fungi and plants form not only GIPC but also GlcCer. SPT, serine palmitoyltransferase; SphK, sphingosine kinase; S1PL, sphingosine-1-P lyase; CerS, ceramide synthase; CDase, ceramidase; SMS, sphingomyelin synthase; SMase, sphingomyelinase; Cerk/ACD5, ceramide kinase; GCS, glucosylceramide synthase; IPCS, inositol phosphoryl ceramide synthase; MIPCS, mannosyl inositol phosphorylceramide synthase; DHSPH, dihydrosphingosine; SPH, sphingosine; PhytoSPH, phytosphingosine; SPH1P, sphingosine-1-P; DHCer, dihydroceramide; Cer, ceramide; PhytoCer, phytoceramide; C1P, ceramide-1-P; SM, sphingomyelin; GlcCer, glucosylceramide; GalCer, galactosylceramide; IPC, inositol phosphorylceramide; MIPC, mannosyl inositol phosphorylceramide; GIPC, glycosyl inositol phosphoryl ceramides.

The biosynthesis of sphingolipids is highly conserved among eukaryotes and starts with the condensation of palmitoyl-CoA and serine to produce 3-keto dihydrosphingosine, a step catalyzed by the enzyme serine palmitoyltransferase (SPT; Hanada, 2003). LCBs can be modified by *N*-acylation, phosphorylation, desaturation, and hydroxylation. Sphingosine is *N*-acylated with C16 or C18 and saturated or (*E*)-Δ3-unsaturated fatty acids by ceramide synthase (CerS), resulting in the formation of dihydroceramide (Vallee and Riezman, 2005). Sphingosine can also be phosphorylated by sphingosine kinases (SphK) to generate sphingosine-1-phosphate (S1P; Futerman and Riezman, 2005). S1P-lyase (S1PL) catalyzes S1P hydrolysis to produce hexadecenal and phosphoethanolamine. Following dihydroceramide biosynthesis, a hydroxyl group is inserted at the C2 position of the fatty acid chain to generate α-OH-dihydroceramide, a step catalyzed by fatty acid 2-hydroxylase. The next step in sphingolipid biosynthesis consists of the C4 reduction in the sphingoid base of α-OH-dihydroceramide by the enzyme sphingolipid Δ4-desaturase, which takes place on the cytosolic face of the endoplasmic reticulum (ER) and generates α-OH-ceramide (Ternes et al., 2002). A double bond between the C8 and C9 positions and a methyl group at C9 are then introduced in the LCB by the enzymes sphingolipid Δ8-desaturase and sphingolipid C9-methyltransferase (SMT), respectively, forming OH-Δ8-9-methyl-ceramide, which is uniquely found in fungi, except for *S. cerevisiae* (Ternes et al., 2011). The Δ8-unsaturated and C9-methylated sphingoid base is characteristic of fungal glucosylceramides (GlcCer) and galactosylceramides (GalCer). The last step of the biosynthetic pathway involves the transfer of a sugar residue from UDP-glucose or UDP-galactose to the ceramide backbone by glucosylceramide synthase (GCS) or ceramide galactosyltransferase, respectively, in the Golgi apparatus (Figure 2; Leipelt et al., 2001; Warnecke and Heinz, 2003).

An alternative route to sphingolipid synthesis starts with the conversion of sphingosine into phytosphingosine by the enzyme sphingolipid C4 hydroxylase (Li et al., 2007). Next, a very long fatty acid chain containing 18, 24, or 26 carbons is amide-linked to phytosphingosine by CerS, forming phytoceramide (Dickson, 2008). Phytoceramide is then used as the substrate for the biosynthesis of complex sphingolipids in the Golgi. The first reaction, catalyzed by IPC synthase (IPCS), involves the transfer of a myoinositol-1-phosphate group from phosphatidylinositol to the C1 hydroxyl position of phytoceramide, generating inositol-phosphoryl ceramide (IPC; Kuroda et al., 1999). Further processing of IPC by glycosyltransferases generates glycosyl inositol phosphoryl ceramides (GIPCs); these anionic glycosphingolipids (GSLs) are found in several fungi and their levels are regulated during morphogenesis (Guimaraes et al., 2014). The glycan moieties of fungal GIPCs show great diversity and complexity, varying among species and dimorphic morphotypes (Takahashi et al., 2009). In the yeast *S. cerevisiae*, IPC can be further mannosylated to form mannosyl inositol phosphorylceramide (MIPC). This step is catalyzed by MIPC synthases, which transfer mannose from GDP-mannose to the inositol moiety. The final step in yeast sphingolipid synthesis involves the formation of mannosyl diinositol phosphorylceramide [M(IP)_2_C] through the inositol-phosphotransferase-catalyzed addition of another inositol-phosphate group onto MIPC (Marques et al., 2018).

Ceramide can be phosphorylated by ceramide kinase (CerK) to form ceramide-1-phosphate (C1P) in both mammals and plants (Liang et al., 2003; Marques et al., 2018). Ceramide is hydrolyzed by ceramidase (CDase) to yield sphingosine, which is phosphorylated by SphK to generate S1P, sphingomyelin (SM), and gangliosides, which are produced specifically in mammalian cells (Marques et al., 2018). SM hydrolysis is catalyzed by sphingomyelinase (SMases) to produce ceramide. SM degradation is a major pathway involved in producing ceramide and in particular S1P, the last sphingolipid before the final stage of sphingolipid degradation (Tan-Chen et al., 2020). Gangliosides are cell type-specific GSLs containing one or more sialic acid (*N*-acetylneuraminic acid) modifications on the sugar chain and are enriched in mammalian brain cells, especially in the cerebral cortex (Sandhoff et al., 2018). Cerebrosides, which can be classified as glucocerebrosides, galactocerebrosides, and zwitterionic GSLs, are mainly composed of one hexose and one ceramide (Nishimura et al., 2017). Cerebrosides are widely produced by plants, fungi, and animals (Traversier et al., 2018). GIPCs are the most abundant sphingolipids in plants and fungi, while SM is the most abundant sphingolipid in animals (Gronnier et al., 2016).

## Role of Sphingolipids in Bacterial Infection

**Figure 3:**
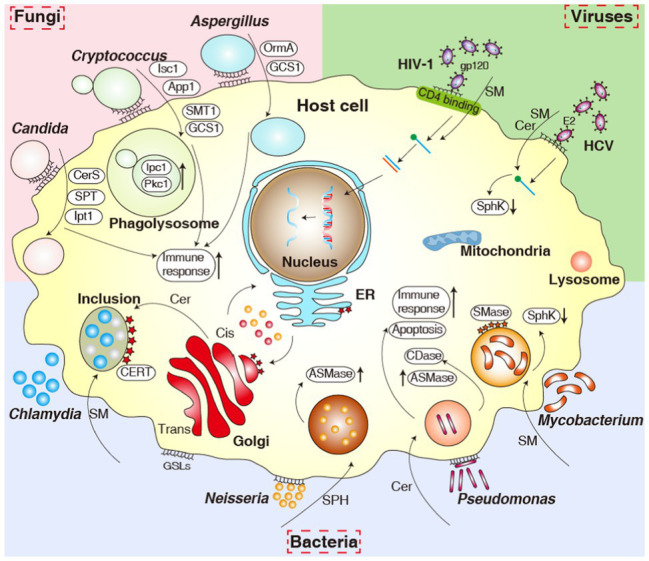
Overview of the various pathogens and their interactions with sphingolipids and sphingolipid signaling pathways. The figure shows bacteria (bottom), fungi (upper left corner), and viruses (upper right corner). ER, endoplasmic reticulum; ASM, acid sphingomyelinase; SphK, sphingosine kinase; SMase, sphingomyelinase; CDase, ceramidase; CerS, ceramide synthase; SPT, serine palmitoyltransferase; Ipt1, inositol phosphotransferase 1; Pkc1, protein kinase C1; Isc1, inositol phosphosphingolipids-phospholipase C1; App1, antiphagocytic protein 1; SMT1, sphingolipid C9-methyltransferase 1; GCS1, glucosylceramide synthase 1; OrmA, orosomucoid protein A; Cer, ceramide; SM, sphingomyelin; SPH, sphingosine; GSLs, glycosphingolipids.

Bacterial pathogens, such as *Sphingobacterium*, *Sphingomonas*, *Bacteroides*, *Prevotella*, *Bdellovibrio*, *Porphyromonas*, *Pedobacter*, *Fusobacterium*, *Cystobacter*, *Mycoplasma*, *Flectobacillus*, and possibly *Acetobacter*, can synthesize sphingolipids (Geiger et al., 2010). Among bacteria that cannot synthesize sphingolipids, most bacterial pathogens, such as *Mycobacteria*, *Pseudomonas*, *Neisseria*, *Helicobacter*, *Chlamydia*, *Legionella*, and *Staphylococcus*, have developed different strategies to manipulate and use host sphingolipids to promote their pathogenicity. In many cases, the attachment and uptake of pathogenic bacteria, as well as bacterial development and survival within the host cell, depend on sphingolipids (Figure 3; Kunz and Kozjak-Pavlovic, 2019). The role of sphingolipids during the establishment of a bacterial pathogenic infection has recently come into focus; below, we discuss how these pathogens use host sphingolipids and their own sphingolipids during the complex steps leading to infection.

## Chlamydiae

Chlamydiae are some of the most widespread bacterial pathogens in the world, costing billions of dollars annually. All Chlamydiae species are obligate intracellular pathogens that primarily grow in host epithelial cells. They alternate between two distinct forms: the infectious extracellular spore-like form called as the elementary body (EB) and the non-infectious but metabolically active intracellular form known as the reticulate body (RB). The EB form transitions to the RB form for replication (Elwell and Engel, 2005). The exit of EBs from the host cell completes the cycle and allows for the infection of neighboring cells. A recent study showed that host cell sphingolipids are essential for chlamydial growth, multiplication, and infection (Kunz and Kozjak-Pavlovic, 2019).

Chlamydial inclusions are membrane-bound compartments that hijack the host exocytic pathways to obtain nutrients, resulting in primary differentiation of the pathogen within the first 1–2 h after organism entry (Moore et al., 2008). Inside a mammalian cell, the inclusion is isolated from the endocytic pathway and is fused with exocytic vesicles containing SM derived from the Golgi apparatus to the plasma membrane (Fields and Hackstadt, 2002). Host-cell-derived SM transiently associates with the inclusion membrane and is incorporated into the chlamydial cell wall. To fuel its expansion, the vacuolar membrane surrounding Chlamydiae receives host-cell-derived sphingolipids by fusing with intercepted ER- and *trans*-Golgi network-derived secretory vesicles. The interception of these vesicles results in the deposition of SM on the inner surface of the inclusion membrane; RBs subsequently attach to and feed from this membrane (Hackstadt et al., 1997). The requirement for sphingolipids by Chlamydiae is initially low but gradually increases as more bacteria replicate and the inclusions expand. The hijacking of host SM stores starts at approximately 20 h after infection, which is consistent with observations of Golgi fragmentation and chlamydial expansion, and continues during persistent infections (Dille et al., 2014). The inhibition of SM biosynthesis in the host cell results in the loss of chlamydial inclusion membrane integrity and early release; host-cell-derived SM is thus necessary for the fusion of chlamydial inclusions and persistent infection (Elwell et al., 2011).

The small GTPase Rab14, a host protein, controls intracellular vesicle trafficking from the Golgi to endosomes and promotes the delivery of SM from the Golgi to chlamydial inclusions, thus influencing *Chlamydia trachomatis* infection (Capmany and Teresa Damiani, 2010). In addition, inhibiting Golgi dispersal during chlamydial infection by depleting Rab6, Rab11, or Rab39a impedes the acquisition of SM by the bacteria and therefore reduces bacterial infectivity (Rejman Lipinski et al., 2009; Kokes et al., 2015; Tudela et al., 2015). *Chlamydia trachomatis* uses the Akt/AS160 (Akt substrate of 160 kDa) signaling pathway to promote the delivery of SM to chlamydial inclusions through Rab14-controlled vesicular transport (Capmany et al., 2019).

Ceramide transfer protein (CERT) is a host protein that transports ceramide from the ER to the Golgi and has been identified as a host cell factor that is specifically recruited to *C. trachomatis* and *C. muridarum* inclusions. Moreover, the *C. trachomatis* inclusion membrane protein IncD specifically interacts with the PH (pleckstrin homology) domain of CERT, suggesting that IncD is involved in the recruitment of CERT to *C. trachomatis* inclusions. Co-transfection studies together with phylogenetic analysis revealed that both the N- and C-terminal regions of IncD are required for binding to the CERT PH domain, allowing *C. trachomatis* to redirect host cell-derived ceramides to inclusions more efficiently (Kumagai et al., 2018). In cells depleted of CERT (*via* short interfering RNAs and siRNAs), CERT was no longer detected on the surfaces of *C. trachomatis* inclusions, which resulted in smaller inclusions than cells treated with control siRNAs (Derre et al., 2011; Elwell et al., 2011). Knocking out *CERT via* genome editing with clustered regularly interspaced short palindromic repeats (CRISPR) and the CRISPR-associated nuclease Cas9 reduced inclusion growth and progeny formation upon *C. psittaci* infection, suggesting that CERT is crucial for the establishment of such infection (Koch-Edelmann et al., 2017).

A temperature-sensitive mutant in *serine palmitoyltransferase* (*SPT*) in the Chinese hamster ovary cell line SPB-1 prevented *C. trachomatis* replication at 39°C due to a deficiency in sphingolipids. However, chlamydial replication was restored by introducing a wild-type copy of the *SPT* gene in the mutant cells, as well as by exogenous treatment with sphingosine and dihydroceramide. These findings imply that host-cell-derived sphingolipids are required for the pathogenesis and intracellular growth of *C. trachomatis* (van Ooij et al., 2000).

## Mycobacteria

Mycobacteria are facultative intracellular bacteria that infect their hosts primarily *via* inhalation. After entering the lung, these bacteria are internalized, primarily by alveolar macrophages, giving rise to various severe human infectious diseases worldwide. The survival of most mycobacteria in the host requires host macrophages to repress phagosome maturation including fusion with the lysosomal compartment, acidification of the compartment, and assembly of membrane actin (Russell, 2006). *Mycobacterium tuberculosis*, the human pathogen that causes tuberculosis, currently infects approximately one-third of the population worldwide. The dynamic interaction between *M. tuberculosis* and human macrophages is crucial for tuberculosis pathogenesis (Kusner, 2005). Many groups have recently demonstrated a critical role for sphingolipids in promoting the intracellular viability of *M. tuberculosis*.

The phagocytosis of live, virulent *M. tuberculosis* results in the inhibition of phagosome–lysosome fusion and the subsequent increase in intracellular survival of the pathogen; in contrast to some types of phagocytosis, the phagocytosis of *M. tuberculosis* is not accompanied by elevated macrophage cytosolic Ca^2+^ levels (Malik et al., 2001). In addition, selective chemical inhibitors of the macrophage SphK block the increase in cytosolic Ca^2+^ levels induced by the presence of killed *M. tuberculosis*, thus preventing Ca^2+^-dependent phagosome maturation (Malik et al., 2003). SphK catalyzes the conversion of sphingosine to S1P to increase cytosolic Ca^2+^ levels by releasing Ca^2+^ from ER stores (Spiegel and Milstien, 2002). Together, these findings suggest that the repression of SphK by live virulent *M. tuberculosis* allows the pathogen to evade Ca^2+^-dependent phagosome maturation and bactericidal activity (Kusner, 2005).

Mycobacteria possess two membranes to protect themselves: the lipid-rich outer membrane and the inner membrane with its complement of specific transporters (Niederweis, 2008). SM, an abundant lipid in the macrophage membrane, is delivered from the outer to the inner phagosome leaflet (Gaus et al., 2005). Rv0888, a cell surface-associated SMase in *M. tuberculosis*, enhances the intracellular replication of the pathogen in human macrophages by using SM as a nutrient pool for carbon, nitrogen, and phosphorus (Griffin et al., 2011; Speer et al., 2015).

When cellular sphingolipid levels were reduced in host macrophages by the addition of the toxin fumonisin B1, the entry of *M. tuberculosis* was much more limited than in control cells, as evidenced by counting colony forming units and fluorescence imaging of the pathogen. By contrast, the same conditions had no effect on the entry of *Escherichia coli*, indicating that host sphingolipids are specifically required for mycobacterial entry (Viswanathan et al., 2018).

## Pseudomonas

Cystic fibrosis (CF) is one of the most common human autosomal genetic recessive disorders. CF eventually leads to the death of the patient and affects approximately 40,000 children and young adults in the United States and 300,000 individuals worldwide (O’Sullivan and Freedman, 2009). This disease is caused by mutations in *Cystic fibrosis transmembrane conductance regulator* (*CFTR*), which lead to the accumulation of viscous mucus at the epithelial surfaces of various organs. Inflammation and chronic lung infections by *Pseudomonas aeruginosa* are observed in over 80% of CF patients (LaBauve and Wargo, 2014). These patients have a defective immune response, including massive levels of proinflammatory cytokines in the airway and sustained inflammatory responses, ultimately resulting in airway obstruction and lung damage (Dechecchi et al., 2011).

Acid sphingomyelinase (ASMase) hydrolyzes SM to ceramide, preferentially at an acidic pH, and can be activated by various bacterial pathogens. Indeed, *P. aeruginosa* infection triggers the activation of ASMase and the release of ceramide within a few minutes after infection in normal respiratory epithelial cells (Grassme et al., 2003). Silencing of *ASMase* (*SMPD1*) by RNA interference (RNAi) and pharmacological inhibition of ASMase activity are associated with decreased inflammatory responses to *P. aeruginosa* infection. Interestingly, the release of ceramide depends on the pathogenicity of the *P. aeruginosa* strain, as ceramide release occurs much more rapidly after infection with very virulent strains (Yu et al., 2009a). During infection, the release of ceramide leads to the formation of large ceramide-enriched membrane platforms, which are involved in bacterial internalization and the induction of apoptotic responses (Grassme et al., 2003; Dechecchi et al., 2011). CFTR-deficient mice infected with *P. aeruginosa* intranasally are much more susceptible to the pathogen than CFTR-positive mice (Di et al., 2006). A defective ASMase pathway after *P. aeruginosa* infection was discovered in both a CF bronchial epithelial cell line and *CFTR* knockout mice, indicating that the induction of ASMase is responsible for modulating the immune response to bacterial infection. In addition, epithelial cells with a mutant *CFTR* gene (IB3-1 cells) recovered their cell death response to *P. aeruginosa* infection when treated with exogenous bacterial SMase (Virella-Lowell et al., 2004; Yu et al., 2009a). Hence, the inhibition of ASMase activity may constitute a novel therapeutic strategy for treating patients with CF.

Host-cell glycosphingolipids are important molecules that contribute to the host specificity and adhesion of *P. aeruginosa* as well as being critical for the internalization of *P. aeruginosa* into nonphagocytic cells (Bentzmann et al., 1996). The gangliotetraosylceramide asialo GM1, containing the GalNAcβ1-4Gal sequence, is an apical receptor that mediates the adherence of *P. aeruginosa* to CF respiratory epithelial cells. This sphingolipid is specifically detected in regenerating respiratory epithelium, a cell type that is more often identified in respiratory epithelial cells from CF patients than in normal respiratory epithelial cells (Imundo et al., 1995). The carbohydrate-binding protein heteromeric lectin LecA is produced by *P. aeruginosa* and localizes to the bacterial outer membrane. LecA has been shown to interact with the host cellular receptor globotriaosylceramide (Gb3), which causes bending of the host plasma membrane and engulfment of *P. aeruginosa*. The LecA–Gb3 interaction is in fact sufficient to induce the initial stages of bacterial entry into host cells (Eierhoff et al., 2014). In addition, the catabolism of GSLs at the host plasma membrane plays an important role in inflammation caused by *P. aeruginosa* infection (Loberto et al., 2014). The inhibition of non-lysosomal glucosylceramidase (GBA2), the enzyme that converts GlcCer to ceramide, is associated with a remarkably reduced inflammatory response in CF bronchial epithelial cells infected by *P. aeruginosa* (Loberto et al., 2014; Schiumarini et al., 2017).

Notably, *P. aeruginosa* also produces and secretes sphingolipid-metabolizing enzymes. Hemolytic phospholipase C (PlcH) hydrolyzes phosphatidylcholine (PC) and SM to generate diacylglycerol (DG) and ceramide, respectively (Luberto et al., 2003b). Host-derived sphingolipids induce the secretion of *P. aeruginosa* CDase, leading to enhanced PlcH-mediated hemolysis (Okino and Ito, 2016). Mutations in *PlcH* in a mouse (*Mus musculus*) model result in decreased bacterial virulence of *P. aeruginosa*, indicating that bacteria use PlcH to metabolize host ceramide, thereby inhibiting the formation of ceramide-enriched rafts (Okino and Ito, 2007). In addition, PlcH-induced hemolysis is significantly attenuated in CDase-deficient *P. aeruginosa* mutants, suggesting that CDase is another *P. aeruginosa* virulence factor that enhances the cytotoxicity of PlcH. Sphingosine promotes the expression of *CDase* through the transcriptional regulator SphR, a member of the AraC family of transcription factors, thereby promoting CDase secretion (Okino and Ito, 2016). Deletion of SphR in *P. aeruginosa* lowers bacterial survival, highlighting the importance of a proper response to host-derived sphingosine (LaBauve and Wargo, 2014).

In *Arabidopsis* (*Arabidopsis thaliana*), *P. syringae* infection induces cell death, generating patches with a water-soaked appearance on susceptible plants, together with a loss of chlorophyll. Loss of function of the ceramide kinase ACD5 increases susceptibility to *P. syringae* infection early in development (Greenberg et al., 2000; Liang et al., 2003). In humans and yeast, orosomucoid proteins (ORMs) negatively regulate SPT, thus playing an important role in maintaining sphingolipid levels. An increase in phytosphingosine contents in plants lacking ORM function leads to increased resistance against *P. syringae* infection (Kimberlin et al., 2016; Li et al., 2016). An *Arabidopsis* mutant of the *CDase* gene *ACER* also displayed increased susceptibility to *P. syringae* (Wu et al., 2015).

## Other Bacteria

During infection by *Neisseria gonorrhoeae*, ASMase is activated to release ceramide, which is a prerequisite for bacterial invasion of distinct nonphagocytic human cell types, including several epithelial cells and primary fibroblasts (Grassme et al., 1997). Moreover, the phagocytosis of *N. gonorrhoeae* mediated by the receptor carcinoembryonic-antigen-related cell adhesion molecule (CEACAM) in human cells results in the rapid activation of ASMase, indicating that ASMase plays a crucial role in *N. gonorrhoeae* internalization (Hauck et al., 2000). With the exception of *N. gonorrhoeae*, the activation of ASMase in different pathogens, such as *E. coli*, *Staphylococcus aureus*, *Listeria monocytogenes*, *Salmonella typhimurium*, and *Mycobacterium avium*, is essential for bacterial infection (Esen et al., 2001; Utermohlen et al., 2003; Falcone et al., 2004; McCollister et al., 2007; Utermohlen et al., 2008). *Neisseria meningitidis* cells expressing *OpcA* showed increased levels of ceramide on their surfaces due to the activation of ASMase in brain endothelial cells. This observation supports the notion that the ErbB2 receptor, which is involved in bacterial uptake, is recruited into ceramide-rich membrane platforms (Simonis et al., 2014). Moreover, ASMase can be exocytosed from *N. meningitidis*-infected brain endothelial cells in a Ca^2+^-dependent manner and modify the outer plasma membrane by promoting the formation of ceramide-rich lipid raft platforms (Peters et al., 2019).

Sphingolipids from *Bacteroides fragilis* contribute to the pathogenesis of mixed infections by directly inhibiting neutrophil function *in vitro* (Kato et al., 2002). *Bacteroides fragilis* takes advantage of its unusual bacterial sphingolipids to initiate signaling cascades that facilitate various bacterial survival strategies (An et al., 2011). Sphingolipids and their metabolites in intestinal bacteria might elicit broader systemic host effects (Heaver et al., 2018). S1P signals are recognized by specific G-protein coupled receptors (GPCRs), which are involved in information transfer across cellular membranes (Nielsen et al., 2017; Marciniak et al., 2018). S1P-like metabolites, which are produced by mammalian gut bacteria, act as specific ligands for mammalian GPCRs (Cohen et al., 2017; Heaver et al., 2018). The role of bacterial sphingolipids in mediating host metabolism by functioning as signaling molecules requires further exploration.

*Shigella flexneri* is less invasive in lipid-deficient cell lines lacking sphingoid-based lipid biosynthesis, demonstrating the involvement of sphingolipids in bacterial entry (Lafont et al., 2002). Sphingolipids are also important in detergent-resistant membranes, where their presence is required to evoke the full secretion-activating capacity of the membranes (van der Goot et al., 2004). Infection of eukaryotic cells with *Legionella pneumophila* triggers a change in sphingolipid metabolism in the host cell and specifically affects sphingosine levels (Rolando et al., 2016a). An in-depth analysis of LegS2, a *L. pneumophila* homolog of the highly conserved eukaryotic enzyme S1PL, found that it is mainly targeted to host cell mitochondria, in contrast to the ER localization of its eukaryote homologs, which demonstrates the remarkable adaptation of LegS2 to the *L. pneumophila* pathogenesis system (Degtyar et al., 2009). LpSPL, another protein with high sequence similarity to eukaryotic S1PL, targets host sphingosine biosynthesis and limits autophagy during *L. pneumophila* infection to promote intracellular survival (Rolando et al., 2016a,b).

*Mycoplasma pneumoniae* infection modulates *SPT* expression or ASMase distribution at certain pathogen titers, suggesting that *Mycop. pneumoniae* infection may influence sphingolipid metabolism in its host, which might be related to its pathogenicity (Yu et al., 2009b). Bacterial-encoded S1PL is a critical virulence determinant of the facultative intracellular bacteria *Burkholderia pseudomallei* and *B. thailandensis*. S1PL-deficient bacterial mutants had a greatly attenuated pathogenicity in murine and insect infection models, highlighting the pivotal role of S1P in host–pathogen interactions (Custodio et al., 2016). The biosynthesis of sphingolipids is also critical for cellular homeostasis and the persistence of the oral anaerobe *Porphyromonas gingivalis*, in addition to their role in pathogenicity (Moye et al., 2016). The obligate intracellular bacterium *Anaplasma phagocytophilum* recruits the GTPase Rab10 to the vacuole in order to reroute exocytic traffic from the sphingolipid-rich *trans*-Golgi network in a guanine-nucleotide-independent manner (Huang et al., 2010). *Anaplasma phagocytophilum* incorporates sphingolipids while reproducing inside the host cell, and sphingolipids are critical for the production of infectious progeny (Truchan et al., 2016). *SphK* knockout mice showed significantly attenuated alveolar bone loss compared to wild-type mice infected with the oral pathogen *Aggregatibacter actinomycetemcomitans*, supporting the notion that S1P plays an important role in inflammatory responses induced by *A. actinomycetemcomitans* (Yu et al., 2016).

## Role of Sphingolipids in Fungal Infection

In recent years, the role of sphingolipids in pathogenic fungi has emerged as a rapidly growing field of study, including their signaling, growth, and virulence (Noble et al., 2010; Liu et al., 2019; Tadini Marilhano Fabri et al., 2019; Zhai et al., 2019; Fernandes and Del Poeta, 2020). Fungi can use their own biosynthesized sphingolipids as signaling molecules, especially to mediate the expression of virulence factors (Liu et al., 2019). At the same time, host sphingolipids and their related metabolic enzymes also play crucial roles in the growth and infection of pathogenic fungi (Shea and Del Poeta, 2006; Singh and Del Poeta, 2011; Figure 3). Here, we review how fungal and host sphingolipids regulate pathogenic fungal pathogenesis.

## Candida

*Candida albicans* is an opportunistic human fungal pathogen that can colonize and invade host tissues when immune systems are weak or when competing flora are eliminated (Wall et al., 2019). This species exhibits a variety of morphologies during growth, including round buds, elongated pseudohyphae, and filamentous hyphae (Berman and Sudbery, 2002) and causes infections ranging from superficial skin infections to life-threatening invasive infections with mortality rates exceeding 50% worldwide (Andes et al., 2012). The filamentous hyphae of *C. albicans*, which play an important role in fungal virulence and pathogenesis, form in a polarized fashion mediated by the actin cytoskeleton (Oberholzer et al., 2006). Pathogenic hyphae are distinguished from budding cells and pseudohyphae by their unique growth pattern and hyphae-specific virulence factors (Nantel et al., 2002).

Sphingolipids and related biosynthetic enzymes contribute to hyphal growth and virulence (Noble et al., 2010). Two CerSs in *C. albicans*, CaLag1p and CaLac1p, produce distinct ceramides with differing fatty acid chain lengths. Inositol-containing sphingolipids produced by CaLag1p are required for polarized growth and hyphal morphogenesis, while CaLac1p is essential for the biosynthesis of GSLs, which play a minor role in morphogenesis (Cheon et al., 2012). Blocking sphingolipid biosynthesis with myriocin, a specific SPT inhibitor, causes a loss of membrane polarization and abnormal hyphal morphogenesis in *C. albicans* (Martin and Konopka, 2004).

The recent screening of a *C. albicans* deletion mutant library for infectivity in a mouse model and for morphological switching and cell proliferation *in vitro* revealed that GlcCer biosynthesis is required for virulence. Interestingly, GlcCer appears to act as a virulence effector independently of morphogenetic switching in *C. albicans* (Noble et al., 2010; Prasad and Singh, 2013). In budding yeast (*S. cerevisiae*), *Inositol Phospho Transferase1* (*IPT1*) encodes a key protein involved in the transfer of a phosphoinositol head group onto MIPC to form M(IP)_2_C (Dickson et al., 1997). Mutants of *C. albicans IPT1* exhibit defects in their ability to form hyphae and reduced adhesion to gingival epithelial cells compared to the wild-type and revertant strains (Prasad et al., 2005; Rouabhia et al., 2011). Moreover, the *Δipt1* deletion mutation prevents the activation of Toll-like receptors as well as the expression of *β*-*defensin*, indicating that the *Δipt1* mutant cannot activate the innate immune defense responses of gingival epithelial cells (Rouabhia et al., 2011). Therefore, *C. albicans Δipt1* mutants are not recognized by gingival epithelial cells and fail to trigger the immune response, so they do not induce inflammation (Rouabhia et al., 2011).

The genetic disruption of *C. albicans* sphingolipid Δ8-desaturase also resulted in decreased hyphal growth rates compared to the wild-type strain on solid medium, pointing to a possible role for Δ8-desaturation of LCBs in ceramides in the morphogenesis of *C. albicans* (Oura and Kajiwara, 2008). Pharmacological inhibition of two enzymes related to sphingolipid biosynthesis, SPT and CerS, resulted in impaired phagocytosis by phagocytes (Bryan et al., 2015; Tafesse et al., 2015). Compromised sphingolipid biosynthesis in mice renders the animals more sensitive to *C. albicans* infection (Bryan et al., 2015; Tafesse et al., 2015). Sphingolipid biosynthesis is therefore critical for phagocytosis and *in vivo* clearance of *C. albicans* (Tafesse et al., 2015). A null mutant in *C. albicans 3-ketosphinganine reductase* (*KSR1*) produces lower levels of IPC than wild type and is defective in the transition from budding to filamentous growth, which is a key virulence determinant (Fornarotto et al., 2006). Recent studies have provided evidence that the modulation of sphingolipid homeostasis in the fungal membrane influences the susceptibility of *Candida* species, including *C. glabrata* and *C. auris*, to antimycotic drugs (Healey et al., 2012; Zamith-Miranda et al., 2019; Shahi et al., 2020). These investigations indicate that sphingolipids also affect drug resistance in *Candida* species.

## Cryptococcus

*Cryptococcus* species can cause life-threatening neurological infections. *Cryptococcus* species typically occupy and replicate in acidic environments, which allows them to live in different environments (Garcia-Rodas and Zaragoza, 2012). *Cryptococcus neoformans*, a common invasive opportunistic pathogen that mainly infects immunocompromised patients, is the most common cause of human fungal meningitis worldwide. Sphingolipid biosynthesis plays a crucial role in cryptococcal virulence (Mor et al., 2015).

IPCS is a fungal-specific enzyme that catalyzes the formation of complex sphingolipids. Downregulation of *IPC synthase 1* conferred a growth defect in *C. neoformans* through a pH-dependent mechanism and significantly lowered the expression of certain virulence traits, indicating that the absence of fungal IPC impairs *C. neoformans* pathogenicity (Luberto et al., 2001). Once *C. neoformans* is inside the phagolysosome, the transcription of *IPC1* increases to support the biosynthesis of complex sphingolipids for infection (Fan et al., 2005). IPCS also produces diacylglycerol, which activates protein kinase C1 (Pkc1) and results in the biosynthesis of the virulence factor melanin (Heung et al., 2004, 2005). Additional studies revealed that IPC1 regulates the expression of antiphagocytic protein 1, a novel fungal factor involved in *C. neoformans* pathogenicity that inhibits the attachment and internalization of fungal cells by macrophages (Luberto et al., 2003a).

GlcCer, a cell surface molecule in *C. neoformans*, is essential for fungal growth in the extracellular environment of the host (Fan et al., 2005). The deletion of *Glucosylceramide synthase1* (*GCS1*) in *C. neoformans* results in the loss of pathogenicity in mouse models (Rhome et al., 2011). A *C. neoformans* mutant strain lacking GlcCer cannot grow *in vitro* at neutral or alkaline pH but grows normally at an acidic pH. The role of GlcCer in *C. neoformans* pathogenicity is to ensure the transition through the cell cycle in alkaline environments with a physiological concentration of CO_2_ (Rittershaus et al., 2006). Along similar lines, inositol phosphosphingolipid-phospholipase C1 (ISC1) from *C. neoformans* hydrolyzes inositol sphingolipids and enhances *C. neoformans* survival in macrophages. ISC1 plays a key role in protecting *C. neoformans* from the hostile intracellular environment of phagocytes to promote fungal neurotropism (Shea et al., 2006; Henry et al., 2011). Disruption of the *C. neoformans* gene *sphingolipid C9 methyltransferase* (*SMT1*), which encodes an enzyme that adds a methyl group to the C9 position of the sphingosine backbone of ceramide, is associated with a more than 80% reduction in virulence compared to the wild type and a complemented strain. Moreover, the structure of GlcCer affects the rigidity of the fungal membrane, indicating that specific classes of sphingolipids are essential in order for *C. neoformans* to establish virulence (Singh et al., 2012). Unsaturation at the C8 position in GlcCer of *C. neoformans* is required for virulence, as the accumulation of saturated sphingosine backbones at C8 leads to higher sensitivity to membrane stressors and higher permeability of the plasma membrane (Raj et al., 2017).

## Aspergillus

*Aspergillus* is a filamentous fungus that commonly cause diseases of the respiratory system. *Aspergillus fumigatus*, for example, is a ubiquitous saprophytic filamentous fungus that causes invasive allergic sensitization and severe asthma in humans, resulting in exceptionally high mortality rates in susceptible populations (Brown et al., 2012). Inhibition of *de novo* biosynthesis of ceramide markedly reduces the inflammatory response triggered by *A. fumigatus* during fungal invasion of the lung (Caretti et al., 2016). The sphingolipid-biosynthesis-related protein OrmA negatively regulates SPT activity. Deletion of *OrmA* in *A. fumigatus* significantly reduced virulence in an immunosuppressed mouse model (Zhai et al., 2019). Similarly, two known GCS inhibitors strongly inhibit germination and hyphal growth of *A. fumigatus*, and neutral lipids exhibit a significantly reduced GlcCer/GalCer ratio in the presence of the inhibitors, suggesting that GlcCer is essential for the normal development of *A. fumigatus* (Levery et al., 2002). The *A. fumigatus* GSL asperamide B directly induces airway hyperreactivity in mice *in vivo* and activates invariant natural killer T (iNKT) cells *in vitro*, indicating that GSLs are involved in chronic respiratory diseases in humans (Albacker et al., 2013). The disruption of the gene *YpkA*, encoding an AGC kinase, in *A. fumigatus* lowers GSL levels, especially in the case of the metabolic intermediates belonging to the neutral GSL branch. YpkA is important for fungal viability *via* its role in regulating GSL biosynthesis, as the *ypkA* mutant strain presents a severe phenotype and a complete absence of conidiation (Tadini Marilhano Fabri et al., 2019).

## Other Fungal Pathogens

The fungal pathogen *Fusarium graminearum* is the most common causal agent of Fusarium head blight, which affects wheat (*Triticum aestivum*), barley (*Hordeum vulgare*), and other small grain crops (Goswami and Kistler, 2004). An *F. graminearum gcs1* mutant exhibits defects in polar growth of fungal hyphae but variable fungal pathogenesis across different hosts (Ramamoorthy et al., 2007). In addition, the *F. graminearum* genome encodes two SMT enzymes, FgMT1 and FgMT2. The *ΔFgmt1* mutant produces C-9-methylated GlcCer to levels comparable to those of the wild type, but the *ΔFgmt2* mutant accumulates only 25% as much methylated GlcCer as the wild type (Ramamoorthy et al., 2009). Similarly, the deletion of *FgMT2* is accompanied by severe growth defects, abnormal conidia, and drastically reduced disease symptoms in wheat and *Arabidopsis* (Ramamoorthy et al., 2009). The acyl-CoA dependent CerS enzyme Bar1 in *F. graminearum* plays a role in mediating cell membrane organization and hence disrupts plant infection. This observation was validated by examining deletion mutants of *Bar1*, which display severely perturbed fungal growth *in vitro* and cannot produce perithecia (Rittenour et al., 2011).

*Paracoccidioides brasiliensis* is a pathogenic dimorphic fungus and the causative agent of paracoccidioidomycosis (San-Blas and Nino-Vega, 2008). The lung fibroblast gangliosides GM3 and GM1 are involved in binding to and infection by *Pa. brasiliensis* (Ywazaki et al., 2011). In the dimorphic phytopathogen *Ustilago maydis*, the inhibition of sphingolipid biosynthesis induces a loss of cell polarity and growth inhibition (Canovas and Perez-Martin, 2009). *Magnaporthe oryzae*, the causal pathogen of rice blast disease, is a major challenge to crop production and global food security. Metabolome analyses of *Ma. oryzae* revealed that sphingolipid biosynthesis is critical during appressorium repolarization and for the pathogenicity of rice blast (Liu et al., 2019).

## Role of Sphingolipids in Viral Infection

Viruses must cross the membranes of their cell targets during the first step of their replication cycle. Sphingolipids are major constituents of membrane lipids whose biogenesis, modifications, and turnover influence membrane dynamics. Their local segregation into lipid rafts also directly affects the biophysical properties of membranes by regulating membrane deformation, vesiculation, and fusogenicity, as well as signal transduction and host cell responses. Therefore, sphingolipids are potential key regulators of the viral replication cycle. Viruses exploit membranes and their components, such as sphingolipids, at all the steps of their replication cycle including attachment and membrane fusion, intracellular transport, replication, protein sorting, and budding. Sphingolipids may serve as receptors, as detailed above for GSLs, and are necessary for infection (Figure 3). For instance, sphingolipids may modulate actin dynamics or they may regulate the lateral or vertical segregation of receptor proteins by altering the biophysical properties of the membrane, thereby providing an environment capable of supporting endocytosis (Schneider-Schaulies and Schneider-Schaulies, 2015).

## Human Immunodeficiency Virus

Human immunodeficiency virus (HIV), in addition to its dedicated protein receptors, also interacts with GSLs. Moreover, the sphingolipid environment directly affects HIV fusion as indicated by the observation that an elevation in dihydrosphingomyelin levels effectively interferes with HIV absorption in tissue culture (Vieira et al., 2011). The HIV envelope protein gp120 binds to the cluster of differentiation 4 (CD4) receptor before entering T lymphocytes, monocytes, or dendritic cells, thereby promoting a conformational change within the gp120 variable loop 3 (V3 loop), which harbors a motif that interacts with the carbohydrate moieties of GSLs. For example, Gb3 acts as a resistance factor against HIV infection. Interference with HIV infection was also observed in cell lines with constitutively high Gb3 levels (Ramkumar et al., 2009). Based on these observations, it was proposed that synthetic Gb3 inhibits HIV infection by increasing Gb3 levels in the plasma membrane (Harrison et al., 2010). Thus, Gb3 can act as an HIV entry receptor and, at elevated levels, it may also prevent infection. Variation in the fatty acid composition of GSLs may be important for the role of Gb3 in verotoxin-induced renal pathology and gp120 binding during HIV infection (Lingwood et al., 2010). Moreover, GalCer binds to HIV to control the early infection-independent phase of HIV transfer to T cells. Thus, GalCer appears to function as an initial receptor for HIV in mucosal epithelial cells (Magerus-Chatinet et al., 2007).

Because all GSLs bind within the V3 loop near the center of the chemokine receptor binding site of gp120, GSLs may regulate HIV fusion and uptake differentially. GSLs and HIV are absorbed onto CD4-negative cells (Magerus-Chatinet et al., 2007). In addition, HIV entry into target cells may affect GSL biosynthesis, as HIV is sensitive to D-threo-1-phenyl-2-decanoylamino-3-morpholino-1-propanol (PDMP), which inhibits the conversion of ceramide into GlcCer by glucosyltransferase (Puri et al., 2004), and to changes in cellular GSL content (Rawat et al., 2004).

The importance of sphingolipids for HIV replication is clear and is especially reflected by the selective enrichment of SM and dihydrosphingomyelin in virion constituents (Brugger et al., 2006). In addition, HIV-chronic obstructive pulmonary disease (HIV-COPD) exhibits a unique plasma metabolome profile that includes sphingolipids and fatty acids. However, additional studies are needed to determine how such metabolic pathways contribute to HIV-COPD and whether therapeutic interventions to alter sphingolipid biosynthesis may reduce the risk of COPD (Hodgson et al., 2017).

## Hepatitis C Virus

Most aspects of the hepatitis C virus (HCV) replication cycle depend on lipid metabolism from the host cell. HCV infection of a host cell consists of three basic steps, entry, replication, and exocytosis; sphingolipids participate in and play key roles in all three steps. The viral surface proteins E1 and E2 form a heterodimer that is thought to be present at the surfaces of HCV particles and is involved in viral entry. Carboxyl-terminally truncated soluble recombinant E2 protein can specifically bind to essential HCV entry factors such as glycosaminoglycans, the membrane protein tetraspanin CD81, and the scavenger receptor class B, type I (BI), thereby allowing HCV to enter hepatocytes and begin to replicate its genome. Ceramide can also regulate the entry and replication of HCV. Higher levels of ceramide within the host cell membrane can reduce CD81 levels in the membrane and thus inhibit HCV entry (Haid et al., 2009).

HCV infectivity is positively correlated with plasma sphingolipid levels, and plasma GSL levels are correlated with viral load in genotype 2 HCV patients (Zhang et al., 2016). Serum sphingolipids have recently garnered more attention because they are currently used as a novel biomarker for hepatic diseases in clinical diagnosis and decision-making (Grammatikos et al., 2016; Muecke et al., 2019). By regulating lipid peroxidation, SphK2 restricts the replication of HCV in Huh-7 human liver cells and primary human hepatoblasts. However, mutations affecting the activity of the NS3-4A protease and the NS5B RNA polymerase, which both contain SM-binding domains, are resistant to the effects of lipid peroxidation. Knockdown of *SphK1* using siRNA inhibited replication of two cell-culture-adapted HCVs in Huh-7.5 cells (Yamane et al., 2014). Differences in HCV genotypes likely contributed to the varying effects of *SphK1* knockdown on HCV replication (Wolf et al., 2019). Moreover, the virus can take advantage of cellular SM, and by binding to SM will activate NS5B RNA polymerase of JFH-1, a genotype 2A strain of HCV, and a genotype 1b RNA-dependent RNA polymerase RdRp, to not only increase the size of cellular lipid droplets but also to raise viral infectivity by decreasing cellular SM levels. Sphingolipids therefore participate in HCV replication in a genotype-specific manner (Weng et al., 2010; Aligeti et al., 2015).

Limiting *de novo* sphingolipid biosynthesis by targeting the activity of the key enzyme SPT acutely affects HCV replication. Studies using chimeric mice with humanized liver cells that were infected with HCV gene type 1a or 1b indicated that the *in vivo* SPT inhibitor polymyxin can significantly reduce serum and liver HCV RNA levels (Umehara et al., 2006). Another study used the SPT inhibitor NA808, which inhibited the replication of all HCV genotypes in chimeric mice with humanized liver cells and significantly reduced viral load (Katsume et al., 2013). In addition, the SPT inhibitor NA255 interferes with the association between sphingolipids and HCV non-structural viral proteins within lipid rafts, thereby also inhibiting HCV replication (Sakamoto et al., 2005). These findings indicate that SPT, a key enzyme regulating *de novo* sphingolipid biosynthesis, contributes to the replication cycle of HCV and that inhibiting SPT activity may prevent HCV replication.

## Severe Acute Respiratory Syndrome Coronavirus 2

The ongoing Coronavirus disease 2019 (COVID-19) pandemic has infected over a 100 million people and has resulted in the death of over 3 million people at the time of writing this review. This disease is brought about by infection with severe acute respiratory syndrome coronavirus 2 (SARS-CoV-2; Wu et al., 2020), which engages a type I transmembrane metallocarboxypeptidase, angiotensin-converting enzyme 2 (ACE2), as the host entry receptor (Hoffmann et al., 2020). SARS-CoV-2 also binds to sialic acids linked to gangliosides, host cell surface sphingolipids (Sorice et al., 2020). Plasma lipidomes of COVID-19 resemble those of GM3-enriched exosomes, with enhanced levels of SMs and GM3s and reduced levels of DAGs. The exosomes of COVID-19 patients with increased disease severity showed increasing enrichment of GM3, suggesting that GM3-enriched exosomes partake in pathological processes related to COVID-19 pathogenesis (Song et al., 2020). To date, several studies have proposed that sphingolipid biosynthesis represents a potential drug target for SARS-CoV-2.

The binding of the virus trimeric spike (S) protein to the cellular ACE-2 receptor is critical for the viral infection cycle (Walls et al., 2020). A ganglioside binding domain has been identified at the tip of the N-terminal domain of the S protein; this domain enables the virus to bind to the lipid rafts of the plasma membrane where the ACE-2 receptor resides (Fantini et al., 2020). Hydroxychloroquine and azithromycin treatment was proposed to synergistically reduce SARS-CoV-2 infection by binding to the gangliosides and ganglioside binding domain, respectively (Andreani et al., 2020). Exogenously applied sphingosine prevents the interaction of the receptor-binding domain of the viral S protein with ACE2, indicating that sphingosine might be used as a novel drug to prevent SARS-CoV-2 infection (Edwards et al., 2020). SARS-CoV-2 infection could impair the activities of enzymes involved in S1P synthesis and the signaling triggered by S1P, thereby affecting the ability of the virus to promote multiple clinical symptoms and the individual response to the virus (Meacci et al., 2020). GCS inhibitors disrupt early stages of SARS-CoV-2 replication and significantly reduced the levels of N protein in infected cells, suggesting that the synthesis of GlcCer is required to support the viral lifecycle (Vitner et al., 2021). Overall, these studies suggest that the sphingolipid pathway represents a potential target for SARS-CoV-2 therapy.

## Concluding Remarks

In recent years, it has become increasingly clear that sphingolipids play important roles in a range of biological processes, from the establishment of infection to defense against pathogens. Sphingolipids and their metabolites not only have dual effects on the microorganism and host in that they assist microbial pathogenesis but also trigger host defenses against the invading pathogen. Many aspects of the immune system may be explained by interactions between host and microbial sphingolipids. Inhibiting microbial utilization of sphingolipids has been demonstrated to limit their pathogenesis. Pathogenic microorganisms, such as bacteria and fungi, therefore face challenges to their survival when sphingolipid levels are low, while at the same time sphingolipids can control autophagy and support pathogen survival, thus ensuring their reproduction.

Compared to studies of mammalian pathogens, studies on sphingolipid metabolism and signaling in phytopathogens are still limited. In plants, the receptors, targets, and mediators of sphingolipid signaling are almost entirely unknown, which provides a wonderful opportunity to study the role of these compounds in plant–pathogen interactions. Some evidence suggests that plant sphingolipids play a key role in biological stress, including resistance to bacterial and fungal pathogens, by initiating programed cell death (PCD). Fungal sphingolipids typically function as elicitors to induce both pathogenesis and plant defense mechanisms, as GlcCer promotes host-dependent virulence. However, little is known about bacterial or other fungal sphingolipids and their roles in plant-pathogens interactions. Whether microbial sphingolipids act as signaling molecules and what their receptors might be have yet to be investigated. The identification of signals that function upstream and downstream from plant sphingolipid metabolites is of great significance for exploring the pathogenesis of plant pathogens, as well as for providing new strategies for disease control in crops.

The SM inhibitor D609, which affects ceramide intake by *C. muridarum*, reduces the propagation of *C. trachomatis* (Elwell et al., 2011; Banhart et al., 2014). Moreover, several short-chain ceramides have antibacterial activity on *N. meningitidis* and *N. gonorrhoeae* (Becam et al., 2017). Thus, a better understanding of microbial pathogenesis involved in resistance mediated by sphingolipids and host sphingolipids may provide a new class of antimicrobial treatments by enabling the proper targeting of sphingolipid pathways, which would complement traditional chemotherapeutic approaches to infectious diseases. Determining the exact mechanisms behind these processes remains a challenge for the future and will be of great value in our fight against pathogens, as antibiotic resistance is on the rise.

## Author Contributions

JW and NY designed, wrote, and edited the article. JW, Y-LC, Y-KL, D-KC, and J-FH gathered the references and participated in writing the article. All authors contributed to the article and approved the submitted version.

## Conflict of Interest

The authors declare that the research was conducted in the absence of any commercial or financial relationships that could be construed as a potential conflict of interest.

## Publisher’s Note

All claims expressed in this article are solely those of the authors and do not necessarily represent those of their affiliated organizations, or those of the publisher, the editors and the reviewers. Any product that may be evaluated in this article, or claim that may be made by its manufacturer, is not guaranteed or endorsed by the publisher.
